# Adenocarcinoma of the sphenoid sinus

**DOI:** 10.11604/pamj.2014.18.284.4416

**Published:** 2014-08-10

**Authors:** Youssef Darouassi, Mehdi Chihani, Mohamed Mliha Touati, Karim Nadour, Haddou Ammar, Brahim Bouaity

**Affiliations:** 1 ENT department, Avicenne Military Hospital, Marrakech, Morocco

**Keywords:** Sphenoid sinus, adenocarcinoma, endonasal surgery, radiotherapy

## Abstract

Adenocarcinomas of the sphenoid sinus are exceptional. In this paper, we report a new case with a review of the literature. Our patient was a 45-year-old man who presented with isolated retro orbital headache. CT and MRI suspected a malignat tumor of the sphenoid sinus. The patient underwent a debulking surgery. The final pathology carried out the diagnosis of primary adenocarcinoma. The patient died several months later from radiotherapy complications. Even if adenocarcinomas of the sphenoid sinus are exceptional, they should be considered in the differential diagnosis of sphenoid sinus masses. The prognosis is poor.

## Introduction

The sphenoid is a central skull base bone. It may be the site of a primary carcinoma or secondary metastasis of breast, lung, kidney, thyroid or prostate carcinoma [1]. Primary carcinomas of the sphenoid sinus are rare. We report a case of a poorly differentiated adenocarcinoma of the sphenoid sinus with review of the literature.

## Patient and observation

**Figure 1:**
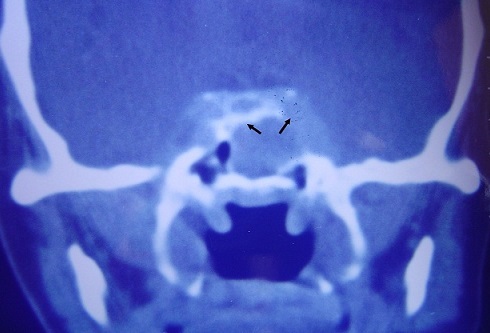
Coronal section CT scan centered on the sphenoid sinus, objectifying process of sinus tissue and bone lysis of the floor of the sella and the left side wall of the sphenoid sinus (arrows)

**Figure 2:**
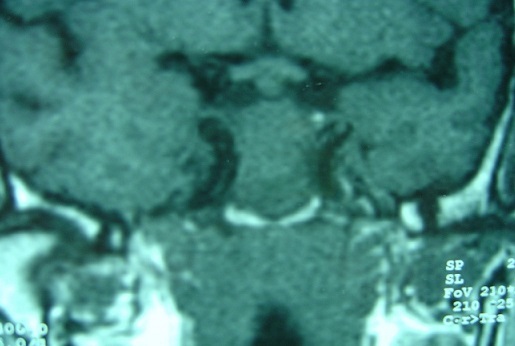
MRI coronal T1 sequence without gadolinium showing a process of the sphenoid sinus tumor with isosignal to brain

**Figure 3:**
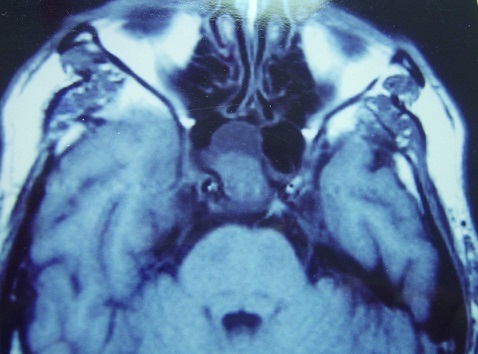
Axial MRI through the sphenoid sinus on T1 with gadolinium, showing a tissue process occupying the sinus with heterogeneous enhancement

**Figure 4:**
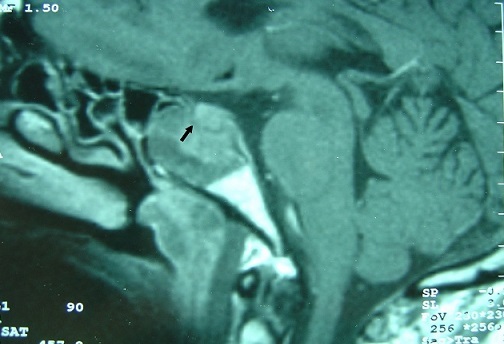
MRI sagittal T1 sequence with gadolinium, highlighting a tissue process with heterogeneous enhancement, occupying the sphenoid sinus and respecting pituitary gland, which is separated by a hyposignal border (arrow)

A 45-year-old man, chronic smoker,with no history of exposure to wood dust or tannin, presented five months prior to hospitalization, isolated retro orbital headache, resistant to analgesics, without fever, neck stiffness, rhinorrhea or epistaxis and without loss of visual acuity. Nasal endoscopy showed healthy nasal mucosa without any suspicious lesion. Neurological examination including cranial nerves showed no cavernous syndrome or sensorimotor deficit. The ophthalmologic examination was normal. Cervical lymph node areas were all free. A CT scan (CT) of the sinuses showed a tumoral process occupying the sphenoid sinus with bone lysis (Figure 1) confirmed with magnetic resonance imaging (MRI) (Figure 2, Figure 3, Figure 4). No pituitary insufficiency or hypersecretion was detected. The staging (chest X-ray, liver ultrasound) did not find any secondary location, which allowed a priori to eliminate a possible metastasis. After consultation with neurosurgeons, a nasal endoscopic approach was decided. At the opening of the sphenoid we discovered a slightly friable and hemorrhagic tumor process. Frozen section examination revealed a malignant tumor. We continued the reduction of tumor volume to the floor of the sella. The suites were simple without leakage of cerebrospinal fluid. The final histological examination of the surgical specimen diagnosed a poorly differentiated adenocarcinoma of the sphenoid sinus. The treatment was completed by an external radiation dose of 50 Gy at 2Gy per day, five days a week for five weeks. The evolution was marked by the appearance of left acute purulent otitis media treated with ciprofloxacin and diabetes insipidus with polyuria syndrome of sudden onset up to 12 liters per day, blood electrolytes showed a serum sodium up to 157mmol by liter and a blood hyperosmolarity. The origin of this central diabetes insipidus was confirmed by the test to desmopressin ( DDAVP), which yielded a concentration of urine. The patient was placed under DDAVP nasal spray two times a day. Local control after radiotherapy showed the persistence of residual tumor extended to the left cavernous sinus. 6 months later, the patient was received in emergency intensive care unit with dehydration and left ophthalmoplegia. He died a few hours after admission.

## Discussion

Primary malignant tumors of the sphenoid sinus are very rare varying from 1 to 2% of sinus tumors according to studies[2, 3]. Adenocarcinoma of the sphenoid sinus is extremely rare[4].

Clinically, the patient could present with isolated chronic headache (as in our case), other symptoms may be part of the clinical picture according the localization and size of the tumor including: diplopia by VI nerve damage epistaxis, orbital pain, cavernous syndrome by No. II, III and VI cranial nerves lesion. The diagnosis can be made at a neurosurgical stage by intracranial extension or the stage of lymph node, bone or lung metastases.

CT and MRI can reveal a tumor of the sphenoid sinus, they sometimes show signs of suspected malignancy. In our patient CT showed bone destruction of the walls of the sphenoid sinus suspecting malignancy.

Nasal endoscopic biopsy and histological study allow the diagnosis. The histological types found in the literature are: squamous cell carcinoma, undifferentiated carcinoma, adenoid cystic carcinomas, adenocarcinomas, non-Hodgkin lymphoma, lympho-epithelioma, lymphosarcoma, papillary carcinoma, malignant adamantimomas, leiomyosarcoma, osteosarcoma and undifferentiated sarcomas[5, 6]. Other rare primary malignant tumors of the sphenoid sinuses are described in the literature including esthesioneuroblastoma [7], malignant melanoma [8], and myxofibrosarcoma [9].

No specific TNM classification exists for the sphenoid sinus [10]. The treatment of malignant tumors of the sphenoid sinus is based on a combination of debulking surgery and radiotherapy [11]. Total resection of these tumors is very difficult due to their topography and proximity to the carotid, optic nerves, cavernous sinus and sella. Radiotherapy alone is considered for inoperable tumors. Chemotherapy is implemented either in combination with radiotherapy or in case of recurrence after radiotherapy [2].

The prognosis of these tumors is characterized by the death of the majority of the patients reported in the literature. This issue occurs more or less rapidly depending on the stage of diagnosis, tumor size, extension to the skull base, histological type, aggressiveness, and complications of therapeutic methods [1].

## Conclusion

The sphenoid sinus may be the starting point for primary malignant tumors of different histological types. The otolaryngologist should be able to make early diagnosis of these tumors before the first warning signs including isolated retro orbital chronic headaches. CT and MRI allow highlighting the process occupying the sphenoid sinus and assess the tumor extension. Biopsy of these tumors is currently facilitated by the endonasal endoscopic surgery, which allows easy access to the sphenoid sinus.
